# Maral Root Extract and Its Main Constituent 20-Hydroxyecdysone Enhance Stress Resilience in *Caenorhabditis elegans*

**DOI:** 10.3390/ijms26083739

**Published:** 2025-04-15

**Authors:** Velislava Todorova, Monika N. Todorova, Martina S. Savova, Kalin Ivanov, Milen I. Georgiev, Stanislava Ivanova

**Affiliations:** 1 Department of Pharmacognosy and Pharmaceutical Chemistry, Faculty of Pharmacy, Medical University of Plovdiv, 4002 Plovdiv, Bulgaria; 2 Research Institute, Medical University of Plovdiv, 4002 Plovdiv, Bulgaria; 3 Laboratory of Metabolomics, Institute of Microbiology, Bulgarian Academy of Sciences, 4000 Plovdiv, Bulgaria; 4 Center of Plant Systems Biology and Biotechnology, 4000 Plovdiv, Bulgaria

**Keywords:** *Caenorhabditis elegans*, adaptogens, *Rhaponticum carthamoides*, 20-hydroxyecdysone, longevity, lifespan, aging

## Abstract

As human life expectancy continues to rise, managing age-related diseases and preserving health in later years remain significant challenges. Consequently, there is a growing demand for strategies that enhance both the quality and the duration of life. Interventions that promote longevity, particularly those derived from natural sources, are popular for their potential to address age-related health concerns. Adaptogens—herbs, roots, and mushrooms—are valued in food science and nutrition for their ability to enhance resilience and overall well-being. Among these, *Rhaponticum carthamoides* (Willd.) Iljin, known as maral root (Russian leuzea), holds a prominent place in Siberian traditional medicine. The root extract, abundant in bioactive compounds such as flavonoids and phytoecdysteroids, is reputed for reducing fatigue, boosting strength, and offering immunomodulatory benefits. However, the effects of the plant extract on lifespan and age-related decline remains poorly studied. This study investigates the effect of maral root extract and phytoecdysteroids—ecdysterone, ponasterone, and turkesterone—on aging using *Caenorhabditis elegans* as a model organism. A sensitive liquid chromatography method with photodiode array detection was developed and validated to quantify the phytoecdysteroids in the extract. Behavioural and stress-response assays revealed that maral root not only extends lifespan but also significantly enhanced healthspan, stress resilience, and fitness in the nematodes. Additionally, treatment with ecdysterone, the most abundant compound in the root extract, improved healthspan by enhancing stress response. These findings underscore the potential of maral root as a natural adaptogen to mitigate age-related decline, providing valuable insights into natural longevity interventions.

## 1. Introduction

The term “adaptogen” was originally used to describe pharmacologically active substances that enhance an organism’s resistance to stress [1]. At the end of the 1960s, Brekhman and Dardimov defined adaptogens as agents that normalize physiological functions by non-specifically boosting resistance to harmful elements—whether chemical, physiological, or physical (commonly referred to “stressors”) [2,3]. The term also refers to certain plants that exert adaptogenic properties. Examples of such plants include *Eleutherococcus senticosus* Max., *Rhaponticum carthamoides* (Willd.) Iljin, *Rhodiola rosea* L., *Aralia cordata* Thunb., and *Shizandra chinensis* (Turcz.) Bail [3,4,5].

In recent classification, adaptogens have been delineated as herbal preparations that enhance attention and endurance during fatigue while alleviating stress-induced impairments and disorders affecting the neuroendocrine and immune systems [4]. Although the precise mechanisms by which plant adaptogens enhance stress resistance remain unclear, they are proposed to interact with key components of the molecular network involved in stress response, many of which also regulate lifespan [1]. Given the well-established correlation between increased stress resilience and extended lifespan, as well as the health benefits attributed to plant adaptogens, they are of considerable interest in longevity research [4,6]. Moreover, their incorporation into everyday nutrition could serve as a key intersection of food science and longevity, highlighting the potential of diet-based approaches for enhancing resilience and extending healthspan.

The concept of aging has evolved to encompass a progressive decline in tissue and organ function, culminating in chronic illnesses such as neurodegenerative diseases, cardiovascular disorders, and cancer, all of which significantly impact human health worldwide [7,8,9]. While numerous factors are believed to contribute to aging, the primary focus of anti-aging research is on enhancing the body’s repair mechanisms and increasing resilience to stressors [6,8,9]. Several model organisms, including *Caenorhabditis elegans*, play a pivotal role in the longevity domain. Nematodes not only share a high degree of genetic homology with humans, particularly in genes known to be critical in aging, but they also exhibit age-related physiological decline, including disruptions in metabolic homeostasis and diminished stress responses [10,11].

In recent years, a variety of strategies, including dietary interventions and pharmacological therapies, have been shown to extend lifespan and promote healthy aging [7,12]. Among these, proper diet and nutrition, abundant in natural products, have emerged as a particularly promising approach [6]. Foods containing compounds such as carotenoids, flavonoids, and vitamins are recognized for their robust antioxidant and cytoprotective activity, which mitigates oxidative stress, delays degenerative processes, and enhances immune and gastrointestinal functions, collectively contributing to improved quality of life [4,6]. Of particular interest are adaptogens derived from medicinal plants, renowned for their capacity to sustain physiological equilibrium and resilience under stress, thus highlighting their potential as pivotal agents in the advancement of healthspan and longevity research [4].

*Rhaponticum carthamoides*, belonging to the Asteraceae family and commonly referred to as maral root or Russian leuzea, is a perennial herb endemic to the alpine and subalpine meadows of Siberia and Altai [13,14]. Distinguished in traditional medicine, it has been valued for its ability to alleviate fatigue and weakness [13,14]. In contemporary applications, extracts derived from the roots and rhizomes of *R. carthamoides*, or specific compounds isolated from these structures, are incorporated into dietary supplements for their adaptogenic and tonic properties. These supplements are employed to promote muscle growth, treat impotence, alleviate mental and physical fatigue, and support recovery following surgery or illness [13].

A diverse array of chemical constituents has been identified in the roots and rhizomes of *R. carthamoides*, with steroids—particularly ecdysteroids—being the predominant class [13,15]. Over fifty distinct ecdysteroids have been characterized in the plant, including 20-hydroxyecdysone (20E, ecdysterone), leuzeasterone, inokosterone, polypodine B, rhapisterone, turkesterone (TU), ponasterone A (PA), and their derivatives [15]. Among these, 20E is the most abundant, with concentrations ranging from 0.049% to 1.74% in the roots [15,16]. Moreover, 20E is one of the primary phytoecdysteroids in certain superfoods such as spinach, quinoa, and asparagus [17]. Research into ecdysterone has primarily focused on its potential performance-enhancing and therapeutic benefits [5]. Key effects of 20E include accelerated adaptation to stress, stimulation of protein synthesis without endocrine disruption, reduction in stress and anxiety, enhancement of antioxidant defences, protection of joint cartilage, neuroprotection, and regulation of metabolic syndrome [17,18,19].

Despite extensive research on the health benefits of 20E across various contexts, the adaptogenic and stress-resilience properties of *R. carthamoides* and its primary compounds, such as ecdysteroids, have not been thoroughly explored in the model organism *C. elegans*. This study aims to quantify some major ecdysteroids in *R. carthamoides* rhizomes and roots extract (RCE) via newly developed high-performance liquid chromatography method with photodiode array detection (HPLC-PDA). Moreover, it addresses the knowledge gap by evaluating whether RCE and 20E can influence stress resistance and overall health in *C. elegans*. Specifically, the impact of these compounds on lifespan, as well as resistance to thermal and oxidative stress, was examined, providing valuable insights into their potential as agents for promoting longevity.

## 2. Results

### 2.1. Chromatography Analysis

#### 2.1.1. Method Development

A rapid and sensitive HPLC-PDA method was developed to precisely quantify 20E, PA, and TU in *R. carthamoides* extract.

**Figure 1:**
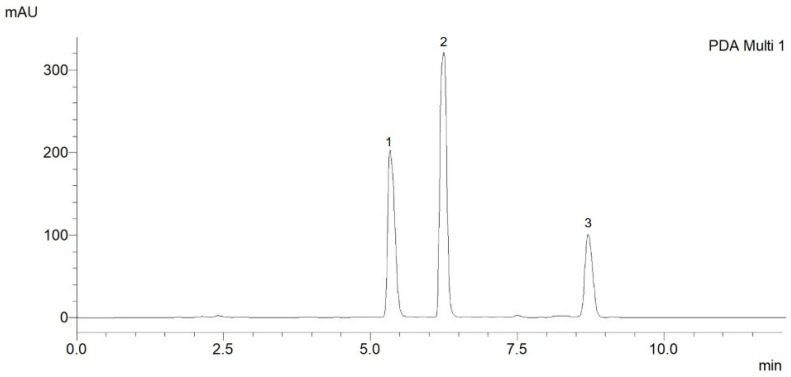
Representative HPLC-PDA chromatogram of the mixture of the three ecdysteroids, where 1—TU (40 µg/mL), 2—20E (40 µg/mL), and 3—PA (40 µg/mL).

The initial phase of method development involved the selection of an optimal mobile phase, comprising water and acetonitrile. Various proportions of acetonitrile and methanol were evaluated to determine the most suitable composition. Both isocratic and gradient elution techniques were tested, with gradient elution demonstrating good peak shape and symmetry. The optimized method resulted in well-resolved chromatographic peaks, with retention times of 5.38 min for TU, 6.38 min for 20E, and 8.63 min for PA (Figure 1). These retention times exhibited excellent reproducibility, with a relative standard deviation not exceeding 0.2%.

System suitability test was conducted to assess the performance of the analytical system and the effectiveness of separation. The assessment included determining the following parameters: retention factor, separation factor, resolution, number of theoretical plates, and symmetry factor. These parameters were calculated according to the United States Pharmacopeia [20]. The obtained data for the suitability of the chromatographic system met the requirements for selectivity and efficiency, indicating good separation and discrimination of the peaks. Due to its accessibility, rapidity, and simplicity, the proposed method was subsequently validated.

#### 2.1.2. Method Validation

1. Linearity

For quantification, an external standard method was employed. The standard solutions, prepared at six concentration levels ranging from 5 to 50 µg/mL, were injected in triplicate to generate calibration curves. The linearity of the developed method was evaluated by correlating the measured peak areas with the known concentrations of the standard solutions. The coefficient of determination (R^2^) was calculated to assess the linearity of the method. The results are presented in Table 1 for linearity, while the calibration curves of 20E, PA, and TU are presented in Figure S1. The obtained results exhibited good linearity, confirming the method’s reliability for the quantification of the target phytoecdysteroids.

2. Accuracy and precision

The accuracy of the proposed method was evaluated using the percentage recovery, calculated as the difference between the mean and assumed true values. For each tested analyte, three quality control levels were utilized: high (40 µg/mL), medium (25 µg/mL), and low (10 µg/mL). The obtained results of the recovery confirmed the high accuracy of the developed method across the specified analytical range. The detailed accuracy assessment results for the tested PEs are presented in Table 2.

The precision of the proposed method was assessed through both intra-day and inter-day evaluations using multiple replicates. Intra-day precision was determined by analyzing freshly prepared solutions at low-, medium-, and high-quality control levels, each measured in five replicates within the same day. Inter-day precision was evaluated by quantifying freshly prepared solutions at the same concentration levels over three consecutive days, with five replicates per level. The results confirmed the high precision of the developed method. Table 3 presents the precision results of the developed method.

The correlation coefficients were close to unity, and the high accuracy percentage and the low values of the standard deviation indicate that the developed method is linear, accurate, precise, and reliable for determining 20E, TU, and PA.

3. Limit of detection (LD) and quantification (LQ)

These were calculated from the linearity data using the standard deviation of a linear response and a slope. The two limits were established as shown in Table 1.

4. Robustness

This was evaluated by assessing the impact of column temperature variations on the chromatographic performance. The column temperature was intentionally varied between 42 °C and 48 °C, and the retention times of the analytes were monitored (Table S1). No significant changes in resolution were observed, indicating that minor temperature fluctuations did not compromise the chromatographic separation, thereby confirming the method’s stability.

To evaluate the stability of the standard solutions, they were stored at a controlled temperature of 2–8 °C for one week. The visual clarity of the solutions was first examined, followed by analysis using the developed method. The stock solutions of ecdysteroids at a concentration of 1 mg/mL were kept for one week at a temperature of 2–8 °C. The stock solutions were then diluted to a concentration within the method range (25 µg/mL). Stability was assessed by comparing the chromatograms of the freshly prepared solutions with those of the stored samples, and the percentage of recovery was calculated (Table S2). The results demonstrated that the samples remained stable throughout the storage period, with no evidence of degradation or alterations in their chemical properties. These findings confirm that the developed method allowed for reliable storage and subsequent analysis of the standard solutions without compromising their integrity.

The correlation factors close to one, the high percentage of accuracy, and the low standard deviation values indicate that the developed method is linear, accurate, precise, and reliable for determining and quantifying 20E, PA, and TU.

**Figure 2:**
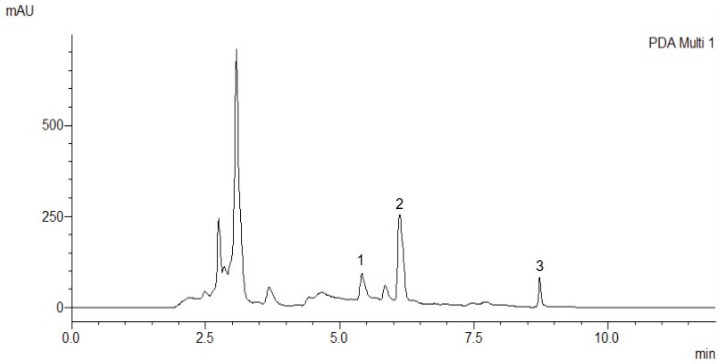
The obtained chromatogram from the analysis of maral root extract, where 1—TU, 2—20E, and 3—PA.

The method was applied to quantify 20E, PA, and TU in maral root extract (Figure 2). According to the calculation through the calibration curve and dilution of the extract, it was determined that 20E was 2.9 mg/g, while PA was 1.3 mg/g, and TU was 1.7 mg/g dry extract. All measurements were performed in triplicate, with a standard deviation not exceeding 2%.

### 2.2. Maral Root Extract Ameliorates Physiological Aging and Increases Lifespan of C. elegans

**Figure 3:**
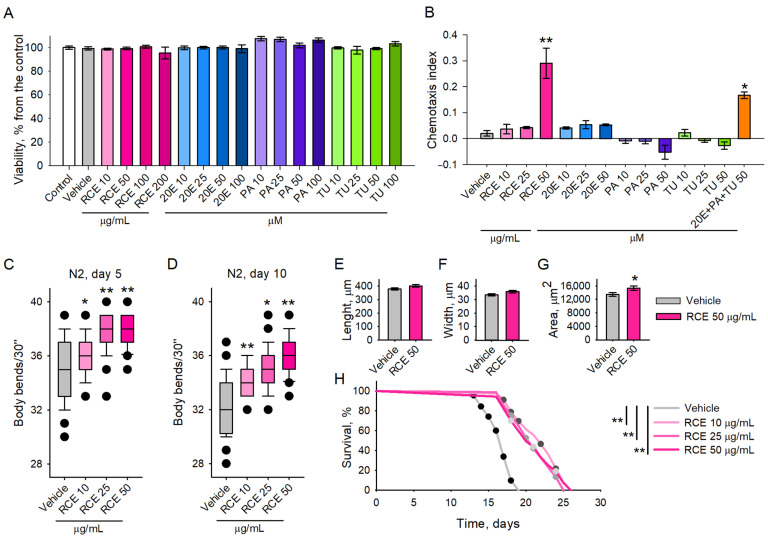
Treatment with RCE stimulates the chemosensory network, improves the motility, and increase the lifespan of *C. elegans*. (**A**) Viability test upon RCE (10–200 μg/mL), 20E, PA, and TU (10–100 μM) in *C. elegans* (n = 1500) compared to control group. (**B**) Chemotaxis assay on *C. elegans* treated with RCE, 20E, PA, TU (10, 25, 50 μM), and combination of 20E, PA, and TU (50 μM; n = 300–600). (**C**,**D**) The mobility of RCE-supplemented worms (10, 25, and 50 μg/mL) on day 5 and day 10, presented as bending movements within 30 sec and compared to vehicle group (n = 45). (**E**) Mean length, (**F**) width, and (**G**) area were measured and analyzed using the WormLab software (version 2023.1.1; n = 110–130). Data for (**A**–**H**) are presented as mean ± SEM. For (**A**–**D**), significance between the groups was assigned as * *p* < 0.05, ** *p* < 0.01 compared to the vehicle group, as determined by one-way analysis of variance (ANOVA). Statistical differences between data in (**E**–**G**) were evaluated by the Mann–Whitney rank sum test with * *p* < 0.05. (**H**) Lifespan of RCE-treated worms (10, 25, and 50 μg/mL), compared to vehicle group, presented as a Kaplan–Meier survival curve (n = 90) expressed as the fraction of survival. The log-rank test was used to evaluate the statistical significance between the experimental groups. For each group, n consisted of a specified number of worms per treatment group, with 3 independent biological replicates.

To evaluate the safety profile of RCE and its phytoecdysteroids, we conducted viability tests in *C. elegans* [21,22]. Worms were pre-treated with RCE at concentrations ranging from 10 to 200 μg/mL and with pure compounds—20E, PA, and TU—at concentrations ranging from 10 to 200 μM for 48 h. The results indicated no toxicity in any of the treatment groups, including those exposed to the extract or the individual compounds (Figure 3A). Based on these findings, we selected concentrations of 10, 25, and 50 μg/mL for RCE and 10, 25, and 50 μM for 20E, PA, and TU for subsequent investigation into their effects on the *C. elegans* chemosensory network.

The results of the chemotaxis assay revealed that only the maral root extract at the highest tested concentration of 50 μg/mL exhibited significant chemoattractant properties (Figure 3B), suggesting that the extract influences sensory perception and feeding behaviour at this dose. At the highest concentration of RCE used in our experiments (50 μg/mL), the extract contained approximately 0.302 µM of 20E, along with 0.171 µM of PA and 0.139 µM of TU (Table S3). For the individual compound treatments and their combination, we used higher concentrations (10–100 μM) than those naturally occurring in the extract to better characterize their biological activity and assess whether any of them could independently replicate the effects observed with the full extract. These elevated concentrations are commonly applied in *C. elegans* assays to reveal functional effects and detect potential biological activity, particularly when a compound may act synergistically within a complex matrix.

Notably, when a mixture of the three main ecdysteroids—20E, PA, and TU—was administered at 50 μM each, a chemoattractant response comparable to that of the full extract was observed (Figure 3B). In contrast, individual treatment with these compounds, even at the same elevated concentrations, did not induce such an effect. These findings support the hypothesis that the observed biological activity of RCE is likely mediated through synergistic interactions among its components, rather than being attributable to a single dominant constituent. Since motility in *C. elegans* is a key indicator of neurological and muscular function as well as overall healthspan, we evaluated locomotor activity by measuring the bending rates of both young (5-day-old) and old (10-day-old) worms.

Our results demonstrated that RCE significantly enhanced locomotion at both time points (Figure 3C,D), suggesting that the extract promotes mobility and may mitigate age-related neurological and muscular decline, particularly in the 10-day-old worms.

Analysis of *C. elegans* morphology using the WormLab (version 2023.1.1, MBF Bioscience, Williston, VT) system revealed that treatment with the maral root extract at a concentration of 50 μg/mL induced a small but insignificant shift in the mean length and width (Figure 3E,F) of the nematodes. In contrast, the mean body area (Figure 3G) of the worms was significantly increased compared to the vehicle group. This alteration was probably attributed to the small changes in the worms’ length and width, which cumulatively contribute to the values of the mean area. These findings, combined with prior observations of enhanced body bending even in aged worms and the chemoattractant properties of the extract, indicate that the extract influences aspects of worm physiology that may contribute to overall healthspan.

Furthermore, we investigated the effect of RCE on the lifespan of *C. elegans* to determine its potential impact on longevity. Our findings indicated a significant, dose-dependent extension of lifespan in the worms treated with RCE (Figure 3H). Specifically, the worms supplemented with the highest concentration of 50 μg/mL exhibited the most pronounced increase in lifespan compared to the control groups, while lower concentrations (10 and 25 μg/mL) also resulted in modest but measurable lifespan extension (Figure 3H). We attribute this effect to the rich composition of RCE, which is abundant in bioactive compounds, including phytoecdysteroids such as 20E, PA, and TU.

### 2.3. The RCE and 20E Treatment Improved Heat Stress Resistance in C. elegans

**Figure 4:**
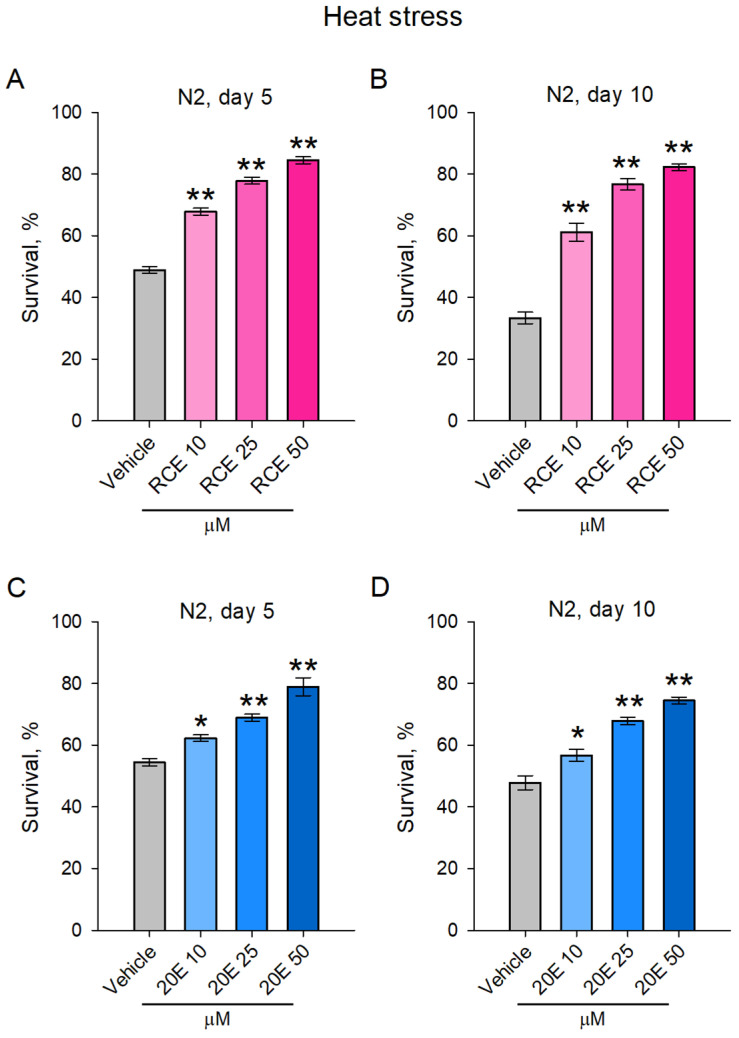
The RCE and 20E treatment improved heat stress resistance in *C. elegans*. Worms were subjected to treatment with RCE (10, 25, and 50 μg/mL; (**A**,**B**) and ecdysterone (20E—10, 25, and 50 μM; (**C**,**D**). Thereafter, both 5-day-old (**A**,**C**) and 10-day-old (**B**,**D**) nematodes were exposed to acute heat stress at 37 °C for 2 h. The viability was monitored after a recovery period of 20 h. The results were represented as percentage survival as mean ± SEM, n = 90 (30 worms per group, 3 biological replicates), * *p* < 0.05, ** *p* < 0.01 (one-way ANOVA).

Aging is commonly associated with increased susceptibility to environmental stressors, including high temperatures, and a diminished capacity to cope with extreme conditions [23,24]. In *C. elegans*, thermotolerance can be used as an indicator of health. Supplementation with natural products may enhance the resistance to stressors, such as heat, which is frequently associated with improved healthspan and longevity [24,25,26]. Adaptogenic plants, such as maral root, are known for their ability to improve an organism’s resilience by adjusting responses to both physical and mental stress, thus enhancing endurance [4]. To investigate whether RCE could improve stress endurance, we subjected both young (5-day-old) and aged (10-day-old) worms to acute heat stress (exposure to 37 °C for 2 h). The results showed that RCE significantly improved thermotolerance in both age groups, with the highest concentration of 50 μg/mL yielding the strongest protective effect (Figure 4A,B). This indicates that the RCE enhanced the worms’ ability to resist heat-induced damage, regardless of age, likely through mechanisms that support cellular stress responses.

Among the various phytoecdysteroids present in maral root, 20E was selected for further investigation in our study because it is the most abundant compound, with concentrations in the roots reported to vary between 0.049% and 1.74%. Moreover, it is widespread in edible plants and dietary supplements. Numerous studies have shown that 20E possesses strong stress-protective effects, including resistance to thermal and oxidative stress [17,27]. At the highest concentration of RCE used in our experiments (50 μg/mL), the extract contained approximately 0.302 µM of 20E (Table S3). This quantification highlighted 20E as the predominant ecdysteroid in the extract, which, combined with its well-documented stress-protective and metabolic effects, supports our rationale for selecting it for further investigation. The results demonstrated that 20E significantly increased heat stress resistance in a dose-dependent manner in both the young (Figure 4C) and aged (Figure 4D) worms. The worms treated with 20E exhibited enhanced thermotolerance when subjected to acute heat stress (37 °C for 2 h). Higher concentrations of 20E led to a more pronounced protective effect in both age groups, suggesting that its ability to improve stress endurance is linked to the dosage administered. Collectively, in both the young and old worms, the 20E- and RCE-treated groups showed improved stress resistance compared to the vehicle groups.

### 2.4. RCE and 20E Enhance Survival in C. elegans Exposed to Oxidative Stress

**Figure 5:**
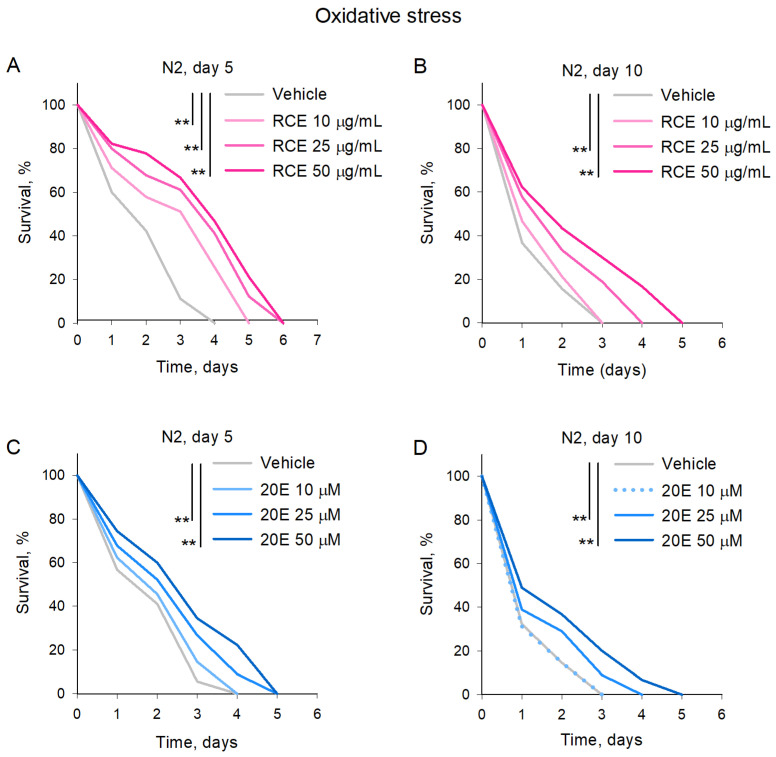
The RCE and 20E treatment improved oxidative stress resistance in *C. elegans*. Wild-type N2 strains of *C. elegans* were subjected to RCE treatment at concentrations of 10, 25, and 50 μg/mL (**A**,**B**) and 20E at concentrations of 10, 25, and 50 μM (**C**,**D**). Both 5-day-old (**A**,**C**) and 10-day-old (**B**,**D**) worms were exposed to paraquat (50 mM). The viability of the worms was monitored at 24 h intervals. Data were analyzed using Kaplan–Meier analysis, and *p* values were calculated using the log-rank test, n = 90 (30 worms per group, 3 biological replicates), ** *p* < 0.01.

To assess the impact of RCE and ecdysterone on oxidative stress response, we subjected *C. elegans* to high doses of paraquat (50 mM) at two distinct time points of their lifespan, i.e., day 5 and day 10. The results showed that both RCE and 20E significantly improved the worms’ resistance to oxidative stress. In the young (5-day-old) worms (Figure 5A,C), treatment with RCE and 20E led to enhanced survival compared to the control group. The highest concentrations of both RCE and 20E provided the most pronounced protective effects. Similarly, in the aged (10-day-old) worms (Figure 5B,D), treatment with RCE and 20E also significantly increased resistance to oxidative stress. The improvements observed in survival were dose-dependent, with the highest concentrations of RCE and 20E offering greater protection. These findings indicate that both RCE and 20E enhance the ability of *C. elegans* to withstand oxidative damage caused by paraquat treatment, suggesting their potential as agents for improving oxidative stress resistance across different life stages.

### 2.5. Rhaponticum Carthamoides Extract Modulates Lipid Metabolism in Glucose-Supplemented C. elegans

Our study investigated whether RCE administration for 48 h influenced lipid metabolism in glucose-fed worms. Given that metabolic dysregulation accelerates aging, interventions that target metabolism are essential for improving healthspan [6]. Glucose supplementation has been shown to induce metabolic stress, simulating aspects of unhealthy diets that lead to lipid accumulation and metabolic disorders [6,22]. By examining the effects of RCE on glucose-fed *C. elegans*, we aimed to determine whether the extract could modulate lipid metabolism under metabolic stress and potentially mitigate aging-related metabolic disruptions.

**Figure 6:**
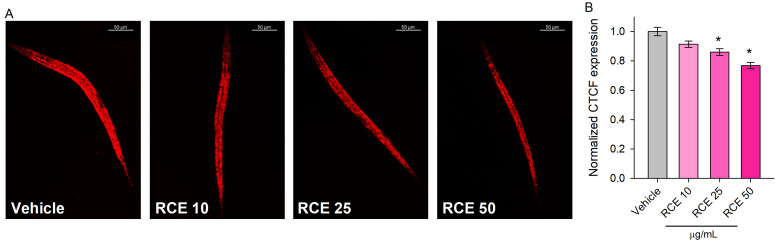
*Rhaponticum carthamoides* extract modulates lipid metabolism in glucose-supplemented *C. elegans*. Representative confocal images at 20× magnification (scale bar of 50 μm) of Nile red lipid staining in wild-type N2 nematodes treated for 24 h with RCE at concentrations of 10, 25, and 50 μg/mL compared to the vehicle (**A**). Quantification of lipid accumulation was performed using corrected total cell fluorescence (CTCF), expressed in arbitrary units (a.u.), for the RCE-treated groups (**B**). Results are presented as mean ± SEM (n = 90; 30 worms per group, 3 biological replicates), * *p* < 0.05, compared to the vehicle control group (ANOVA on ranks).

The results revealed a significant decrease in lipid accumulation in *C. elegans* treated with 50 and 100 μg/mL RCE (Figure 6A,B).

This reduction in lipid content underscores RCE’s role in modulating lipid homeostasis and triglyceride accumulation. These findings enhance our understanding of RCE’s impact on lipid metabolism, highlighting its potential as a modulator of metabolic processes in *C. elegans*. The results suggest that RCE can effectively reduce lipid storage, offering insights into its broader physiological effects and potential mechanisms of action.

## 3. Discussion

Adaptogenic plants and their bioactive compounds have played a crucial role in supporting human health and resilience to stress throughout history [5,28]. These plants, characterized by their capacity to modulate stress responses and restore homeostasis, continue to be a focal point of research into natural interventions for healthspan and longevity [1,4,6]. Prominent examples of adaptogenic plants include *Panax ginseng* C.A.Mey., *Withania somnifera* Dunal, *R. rosea*, and *R. carthamoides* [3,5,29,30,31]. Each of these plants is rich in bioactive compounds such as ginsenosides, withanolides, rosavins, and phytoecdysteroids, which contribute to their adaptogenic properties [15,32]. Additionally, unlike synthetic pharmaceuticals, adaptogenic plants exert broad, non-specific effects on the body, enhancing resilience to physical, chemical, and biological stressors. For instance, 20E found in maral root is associated with increased stress resistance, improved energy metabolism, and enhanced recovery in vitro as well as in vivo [18,33,34]. The global market for adaptogenic products is expanding rapidly, reflecting their popularity as natural alternatives for promoting stress resilience and well-being. This highlights the necessity for developing advanced, highly sensitive methods for the rapid screening of the chemical composition of plant extracts, as well as for the comprehensive investigation of their potential bioactivities. Despite the numerous analytical methods developed for the analysis of ecdysteroids and their diversity, high-performance liquid chromatography remains the preferred choice for routine quantitative analysis of ecdysteroids [35]. It offers high sensitivity, reproducibility, and detailed chromatographic profiles, making HPLC essential for quality control [36]. Various liquid chromatographic methods with PDA or mass spectrometry detection and quantification have been applied to ecdysteroid analysis, including studies on *Sida tuberculata* R. E. Fries, Zuo Gui Wan, and other plant extracts [37,38,39], as well as the quantification of ecdysterone in dietary supplements [40,41] and in human serum [42]. Moreover, various solvent systems have been explored for ecdysteroid analysis, including cyclohexane, *n*-butanol, water, ethyl acetate, and chloroform-based mixtures [35]. Some methods require acidic mobile phases [43]. The HPLC-PDA method developed in this study for the quantification of 20E, TU, and PA demonstrates high accuracy, repeatability, and suitability for routine quality control and research applications. HPLC-PDA methods for the simultaneous analysis of 20E, TU, and PA are limited. The method developed in this study is characterized by a faster analysis and the use of non-toxic solvents, and it demonstrates a lower LD and LQ compared to some other existing methods [37,38]. Additionally, the method is user-friendly, time-efficient, and eliminates the need for derivatization or complex sample preparation. Furthermore, the absence of aqueous-phase acidification simplifies the analysis and reduces the risk of potential equipment degradation. While liquid chromatography with mass spectrometry is the preferred method for ecdysteroid analysis due to its high sensitivity and specificity, HPLC-PDA remains a cost-effective and practical alternative for routine analysis. Its simplicity, minimal sample preparation, and lack of ionization or mass spectral interpretation make it more accessible for standard laboratories [35]. While HPLC-PDA methods offer several advantages, they also present certain limitations. Plant extracts are inherently complex mixtures, and their analysis can be challenged by co-elution and the difficulty of separating structurally similar compounds. Therefore, it is advisable to complement HPLC-PDA analyses with liquid chromatography with mass detection techniques [35,44]. In future studies, we intend to further develop the current method using advance detection in order to achieve a comprehensive confirmation of the extract’s component profile.

In the context of aging and healthspan, adaptogenic plants have gained recognition for their ability to modulate key biological pathways linked to stress responses, energy metabolism, and cellular repair mechanisms [1,3,45]. These properties make them particularly relevant in addressing age-related declines in physiological function. Research suggests that adaptogens may support longevity by improving mitochondrial function, enhancing antioxidant defences, and modulating inflammatory pathways. For example, *R. rosea* has been shown to improve endurance and cognitive performance under stress [46,47], while *W. somnifera* exhibits neuroprotective effects and may mitigate anxiety and depression [29,48,49]. Additionally, aging is commonly associated with increased susceptibility to environmental stressors, including high temperatures, and a diminished capacity to cope with extreme conditions [9,23]. In *C. elegans*, thermotolerance is often used as an indicator of health, and it can be modulated by supplementation with natural products. Enhanced resistance to stressors, such as heat, is frequently linked to improved healthspan and longevity [11,17,24]. Adaptogenic plants, like maral root, are known for their ability to improve an organism’s resilience by adjusting responses to both physical and mental stress, thus enhancing endurance [1,31,33,50].

Given these promising attributes, the current research aimed to investigate the effects of maral root extract and its primary bioactive components, phytoecdysteroids, on key aspects of healthspan and longevity. Utilizing *C. elegans* as a model organism, we examined the impact of RCE on locomotion, stress resistance, lipid metabolism, and lifespan, as well as its ability to influence morphology and sensory behaviour. This study sought to expand the understanding of maral root’s adaptogenic properties and its potential role in modulating age-associated physiological decline.

We observed a significant increase in locomotor activity in worms treated with RCE, with the most prominent effects seen in the older worms. This is in line with other studies suggesting that plant extracts with a rich profile of bioactive compounds can mitigate age-related declines in neuromuscular function [25,51,52]. Additionally, the enhanced locomotion in the RCE-treated worms may have resulted from the modulation of neurotransmitter systems or improvements in muscle function. RCE may enhance neuromuscular signalling or increase the health of muscle cells, thus preventing the usual age-associated motor impairments in *C. elegans* [10,11]. Previously, it was suggested that *R. carthamoides* extract promotes muscle protein synthesis, as its combination with *R. rosea* extract improved muscle protein synthesis and power performance in a murine model [30]. Furthermore, increased motility, as evidenced by enhanced bending even in older worms, indicates greater neuromuscular efficiency, which could correlate with healthier internal tissue states and a possible delay of the onset of age-related neuromuscular decline [11]. This effect could be due to the antioxidant or anti-inflammatory properties of the phytoecdysteroids in RCE, which are known to support cellular integrity and function during aging [17].

The chemotaxis behaviour of *C. elegans* provides valuable insights into the neurosensory system, reflecting feeding preferences and the ability to avoid harmful substances [6,21,25,53,54]. In this context, the chemoattractant properties observed with RCE (50 μg/mL) suggest a potential role in modulating sensory-driven behaviour [53,54]. The enhanced chemotactic response seen only when the major ecdysteroids (20E, PA, TU) were applied in combination, but not individually, further supports the idea that the biological effects of RCE may arise from interactions between multiple components. This underlines the importance of considering the extract as a complex mixture, where synergistic effects may be critical for its overall activity.

A notable observation was the increase in the mean body area of the worms treated with RCE, without any corresponding changes in length or width. Given the rigidity of the *C. elegans* cuticle, which limits lateral expansion [17,55,56], this finding may suggest potential internal physiological changes [54]. This may be linked to improved cellular health, possibly through enhanced cell signalling, protein synthesis, or stress resistance pathways [57]. However, in the absence of structural imaging or additional supporting data, this observation should be interpreted with caution. Further studies would be necessary to determine whether the effect reflects a reorganization of internal structures or other physiological processes. In contrast, the chemoattractant properties of the extract suggest that it interacts favourably with neuronal and metabolic pathways, potentially influencing systemic energy allocation and growth patterns.

The dose-dependent effect of RCE on lifespan in our study mirrors findings from other longevity studies on *C. elegans*, where treatment with extracts from adaptogenic medicinal plants resulted in lifespan extension [25,51,52,58,59]. We found that RCE significantly extended lifespan in a dose-dependent manner, with the highest concentration (50 μg/mL) showing the most pronounced effect. This effect may be mediated by the combination of the antioxidant and stress-protective properties of RCE, which have been shown to activate cellular maintenance and repair mechanisms [31,50,60]. This hypothesis is further supported by the improvement in heat stress tolerance observed in both the young and old worms treated with RCE. Studies on natural products and their constituents, including *R. rosea* and 20E, have demonstrated similar effects, enhancing thermotolerance by modulating cellular stress response mechanisms in *C. elegans* and mice [18,32,60]. The ability of RCE to improve thermotolerance, even in aged worms, suggests that it may enhance cellular protective mechanisms, such as heat shock proteins or mitochondrial function [26]. In addition to thermotolerance, both RCE and 20E significantly improved resistance to oxidative stress. The role of ecdysteroids in modulating oxidative stress responses has been well documented, particularly through their ability to enhance antioxidant defence and support mitochondrial function [6,50]. Additionally, studies suggest a role of *R. carthamoides* in energy metabolism and that it decreases oxidative stress by activating the SIRT6/Nrf2 signalling pathway, protecting against myocardial injury; also, the extract of transformed maral roots possesses antioxidant properties that protect CHO cells from oxidative stress [34,35]. Our findings that RCE and 20E improved survival in worms subjected to paraquat-induced oxidative stress further underscore the potential of RCE and 20E alone as stress-protective agents, with implications for aging and age-related diseases.

Adaptogenic plants and their primary constituents are frequently associated with the regulation of metabolism, improved stress resistance, and potential lifespan extension [4]. Our study demonstrates that RCE significantly reduced lipid accumulation in *C. elegans*, particularly at higher concentrations. This finding aligns with the growing body of the literature indicating that adaptogenic plants and phytoecdysteroids can modulate lipid metabolism [33,34,60]. For instance, an extract from *W. somnifera* reduced fat accumulation in *C. elegans* by regulating the ortholog of the human stearoyl-CoA desaturase enzyme [60,61]. Additionally, our previous study demonstrated that RCE and 20E stimulated lipolysis and decreased fat accumulation in human adipocytes, possibly by modulating lipid breakdown pathways [33]. Consequently, the reduction in lipid accumulation observed in this study may reflect a broader effect on metabolic homeostasis, contributing to the overall health benefits of RCE. Furthermore, the observed reduction in lipid accumulation, along with lifespan extension and increased body area, may signify an enhancement of metabolic efficiency [54,57,61,62]. The energy spared from lipid storage could be redirected toward structural growth and maintenance, resulting in a more robust body morphology [22,59,62].

Collectively, our findings suggest that RCE and its constituents may exert their effects through a complex and potentially synergistic interaction with various metabolic and stress response pathways, contributing to improved health and longevity. While the extract’s overall effect appears to result from its multicomponent composition, 20E in particular demonstrates promising potential in modulating stress responses. Our study provides both a quantitative characterization of the extract and insights into its impact on healthspan in *C. elegans*, supporting its relevance as a natural modulator of aging-related processes.

## 4. Materials and Methods

### 4.1. Materials

The reference standards—20E (molecular weight: 480.64 g/M; purity: HPLC ≥ 95%, #89651) and TU (molecular weight: 496.6 g/M; purity: HPLC ≥ 95%, #85781)—were purchased from PhytoLab GmbH & Co. KG, Vestenbergsgreuth, Germany. The ponasterone A reference standard (purity: HPLC ≥ 95%, #16386; molecular weight: 464.6 g/M) was obtained from Cayman Chemical, Ann Arbor, MI, USA. Analytical-grade acetonitrile was provided by Merck KGaA (Darmstadt, Germany). Nematode growth medium (NGM; #MBS652667) was purchased from MyBiosource Inc. (San Diego, CA, USA). Agar powder (#05039), LB broth Lennox (#L3022), M9 minimal salts (#M6030), 3-(4,5-dimethylthiazol-2-yl)-2,5-diphenyl tetrazolium bromide (MTT; #M2128), fluoroshield histology mounting medium (#F6182), Nile red (NR, #72485), and paraquat (purity ≥ 98%) were purchased from Sigma-Aldrich (St. Louis, MO, USA).

### 4.2. Plant Material and Extraction

The dried rhizomes and roots of *R. carthamoides* were purchased from Russia and identified by the Medical University of Plovdiv, Department of Pharmacognosy and Pharmaceutical Chemistry, in accordance with the Russian Pharmacopoeia. The plant material was freeze-dried and pulverized. The extraction procedure used was previously described in detail by Todorova et al., 2023 [33]. Briefly, the pulverized plant material was subjected to ultrasound-assisted extraction with 50% aqueous methanol at 20 °C for 30 min. The obtained extract was filtered, concentrated at 40 °C using a rotary vacuum evaporator, and finally freeze-dried. The RCE was stored at −20 °C prior to further analysis.

### 4.3. HPLC-PDA Analysis

#### 4.3.1. Preparing of Standard and Test Solutions

The stock solutions of the standard substances 20E, PA, and TU were prepared in acetonitrile/water (50:50, *v*/*v*) at a 1 mg/mL concentration. For better dissolution, an ultrasonic bath was used (Bandelin, Berlin, Germany). Before use, the solutions were stored in brown vials protected from light at 4 °C for one week. Working standard solutions were diluted to series of concentrations with acetonitrile/water (50:50, *v*/*v*). For the quantity determination of ecdysteroids, a stock solution from lyophilized extract was prepared in acetonitrile/water (50:50, *v*/*v*) at a 1 mg/mL concentration. Subsequently, it was diluted according to the concentration range of the developed method and filtered through a 0.45 µm syringe filter (Isolab, Eschau, Germany).

#### 4.3.2. Instrumentation

The method was developed using LC40-PDA (Shimadzu, Kyoto, Japan). A C-18 column (150 mm × 4.6 mm, 3 μm), Shim pack (Shimadzu, Kyoto, Japan), was used for the separation.

#### 4.3.3. Chromatographic Conditions

Gradient elution was employed to enhance the separation. Water (A) and acetonitrile (B) comprised the mobile phase with the following conditions: Initially, the flow rate was set to 0.4 mL/min with a composition of 80% A and 20% B. At 4 min, the flow rate increased to 0.5 mL/min, and the proportion of B increased to 50% B. At 7 min, the proportion of B decreased to 40%, and at 10 min, the composition returned to its initial composition. The total run time for the analysis was 12 min. Throughout the analysis, a column temperature of 45 °C was maintained. The injection volume of the sample was 10 μL. Ultraviolet–visible spectra were recorded in the 190–800 nm range, and chromatograms were acquired at 242 nm. The LabSolutions software Available online: https://www.shimadzu.com/an/products/software-informatics/software-option/labsolutions-cs/index.html (accessed on 18 March 2025) was used to gather and analyze the data (Shimadzu Kyoto, Japan).

#### 4.3.4. Validation of HPLC-PDA Method

The developed method was validated according to the International Council for Harmonisation of Technical Requirements for the Registration of Medicinal Products for Human Use with the following validation parameters: linearity, range, precision, accuracy, limits of detection, limits of quantification, and robustness [63].

### 4.4. Caenorhabditis elegans Maintenance and Treatment

The Bristol wild-type strain N2 used in this study was obtained from the Caenorhabditis Genetic Center at the University of Minnesota, USA, which is supported by the NIH Office of Research Infrastructure Programs (P40 OD010440). The worms were maintained under standard conditions on NGM plates, with *Escherichia coli* OP50 serving as a food source. A synchronized population of worms was prepared using a hypochlorite bleaching method [21]. All the experimental treatments were mixed into *E. coli* OP50, which was heat-inactivated at 65 °C for 30 min and concentrated tenfold. The experimental groups included a control group fed only heat-inactivated *E. coli* OP50; a vehicle group containing 0.2% DMSO (final concentration); groups treated with maral root extract (RCE) at final concentrations of 10, 25, 50, 100, and 200 μg/mL; and groups treated with the pure compounds—20E, PA, and TU—at final concentrations of 10, 25, 50, and 100 μM. All experiments were performed in triplicate, with a new generation of worms used for every replicate. A 48 h MTT assay was conducted on synchronized L1-stage worms to assess viability under treatment conditions. The worms were exposed to RCE, 20E, PA, or TU, washed with M9 buffer, and transferred to a 96-well microplate in triplicate. MTT reagent (3-[4,5-dimethylthiazol-2-yl]-2,5-diphenyltetrazolium bromide) was added, and the plates were incubated for 4 h. Formazan crystals were solubilized with DMSO, and the absorbance was measured at 570 nm using a microplate reader. The assay was used as an initial evaluation of the compound effects. This assay served as an initial step to assess compound tolerability in *C. elegans* [21].

### 4.5. Locomotion Assay

For the locomotion assay, synchronized late-L4 larvae were transferred to NGM plates and exposed to RCE at concentrations of 10, 25, and 50 μg/mL and 20E at concentrations of 10, 25, and 50 μM. The start of treatment was designated as day 0 of the lifespan, and locomotion was specifically assessed on days 5 and 10 of the worms’ lifespan. The bending movements of the worms were evaluated following established protocols [21,25]. At each time point, worms from all experimental groups were randomly selected and placed in a drop of M9 buffer on a clear NGM plate with a 30 s acclimatization period. The number of body bends within a 30 s interval was recorded using a KERN & SOHN GmbH (Balingen, Germany) stereomicroscope. The assay was performed in triplicate, with at least 15 worms per group serving as representative samples.

### 4.6. Lifespan Measurement

The lifespan assay was performed as previously described [21]. Synchronized late-L4 larvae were transferred to NGM plates seeded with heat-inactivated *E. coli* OP50 and treated with RCE at final concentrations of 10, 25, and 50 μg/mL. This time point was designated as day 0 of the lifespan. Worm survival was monitored daily, and individuals unresponsive to mechanical stimulation were recorded as dead. The assay was performed in triplicate, with 30 worms per group.

### 4.7. Chemotaxis Assessment

The chemotaxis assay was performed as described by Mladenova et al., 2023 [62]. A petri dish was divided into four quadrants, with one or two quadrants designated for test samples and the others for controls. A 2 μL volume of each sample was added to the designated quadrants. Approximately 100–150 L4 nematodes, suspended in 2 μL of M9 buffer, were placed at the center of the dish. After a 1 h incubation at 20 °C, the dish was cooled to 4–6 °C for 30 min to immobilize the nematodes. Worms in each quadrant were then counted, and the chemotaxis index (CI) was calculated using the following formula: CI = (Quadrant test area 1 + Quadrant test area 2) − (Quadrant control area 1 + Quadrant control area 2)/Total number of nematodes.

### 4.8. Nile Red Staining

Following a 24 h treatment with RCE at the specified concentrations, approximately 1000–1500 L4 larvae per group were collected. Staining was performed as outlined in Todorova M.N. et al., 2024 [25], using Nile red dye. Imaging was conducted using the Stellaris 5 confocal system with an inverted DMi8 microscope (Leica, Wetzlar, Germany). Quantification of fluorescence intensity was carried out using the ImageJ software (version 1.54), and the results were expressed as the normalized corrected total cell fluorescence (CTCF) in arbitrary units (a.u.). To calculate the CTCF for each image, the background area was multiplied by the background mean fluorescence, and this value was subtracted from the integrated density of the worm. Finally, the CTCF values of the experimental groups were normalized against the CTCF of a glucose-supplemented vehicle group.

### 4.9. Heat Stress

The heat stress assay was conducted as previously described [25]. Synchronized late-L4 larvae were transferred to NGM plates and treated with RCE at concentrations of 10, 25, and 50 μg/mL or 20E at concentrations of 10, 25, and 50 μM. Treatment began on day 0 of the lifespan and continued for the specified duration. For each group, a minimum of 30 animals were used, and the experiment was repeated independently at least three times. Heat stress was induced on both the 5th and 10th days of the worms’ lifespan by incubation at 37 °C for 2 h, followed by a 20 h recovery period. After recovery, survival was assessed by gently touching the worms with a platinum wire to identify non-responsive individuals, which were recorded as dead.

### 4.10. Oxidative Stress

The oxidative stress assay was performed following a previously established protocol [21,64]. Briefly, paraquat was mixed with NGM media to a final concentration of 50 mM, stirred, and then poured onto plates. Synchronized late-L4 larvae were transferred to NGM plates and exposed to RCE at concentrations of 10, 25, and 50 μg/mL or 20E at 10, 25, and 50 μM. Treatment was initiated on day 0 of the lifespan and continued for the indicated periods. At least 30 nematodes pre-treated with RCE or ecdysterone were transferred to fresh NGM plates containing 50 mM paraquat on both the 5th and 10th days of their lifespan. Survival was monitored at 24 h intervals until all worms had died. Each experimental group consisted of at least 30 nematodes, and the experiment was independently repeated three times. The results were presented as percentage survival at each time point.

### 4.11. Analysis of Body Morphology

Analysis of *C. elegans* body size, including mean length, width, and area, was conducted using the WormLab system (MBF Bioscience, Williston, VT, USA) as previously described [65,66]. Late-L4-stage larvae pre-treated with the extract for 24 h were observed. Each video was recorded for 1 min and subsequently analyzed with the WormLab software (version 2023.1.1, MBF Bioscience, Williston, VT, USA).

### 4.12. Statistical Analysis

Statistical analyses were performed in SigmaPlot version 11.0 from Systat software GmbH (Erkrath, Germany), and the data were represented as mean ± SEM. Variations between the experimental groups were calculated by one-way ANOVA followed by Tukey’s post hoc test. The level of statistical significance was set at * *p* < 0.05 and ** *p* < 0.01. Non-parametric methods (ANOVA on ranks or the Mann–Whitney rank sum test) were applied when normality was violated. The Kaplan–Meier survival curves were used to plot the data from the oxidative stress assay and lifespan assessment. The log-rank test was used to assess statistical significance (* *p* < 0.05) between the different groups compared, and the Holm–Sidak correction method was applied for multiple pairwise comparisons of the survival curves. The experimental data presented are representative of at least three independent biological experiments.

## 5. Conclusions

In the current study, a reliable and sensitive HTLC-PDA method was proposed for the simultaneous identification and quantification of 20E, PA, and TU in RCE. Moreover, the observed effects of RCE on lipid reduction, motility, and lifespan extension in *C. elegans* underscore its potential as a multifaceted promoter of health span. Our findings provide strong evidence for the protective effects of RCE and 20E against stress and highlight their therapeutic potential in managing oxidative damage while promoting longevity. Further studies are needed to elucidate the specific molecular mechanisms underlying RCE and 20E and to explore the broader applications of these compounds as natural interventions for enhancing longevity and addressing age-related health challenges.

## Figures and Tables

**Table 1:** Evaluation of linearity, limit of detection (LD), and limit of quantification (LQ) of the developed method for detection and quantification of 20E, PA, and TU.

Parameter	Turkesterone	20-Hydroxyecdysone	Ponasterone A
Linear regression line	y = 46,690x + 173,760	y = 76,488x + 154,533	y = 26,176x + 97,062
R^2^	0.9998	0.9999	0.9998
LD	1.51 µg/mL	1.22 µg/mL	1.57 µg/mL
LQ	4.58 µg/mL	3.7µg/mL	4.74 µg/mL

**Table 2:** Evaluation of the accuracy of the developed HPLC-PDA method for the detection and quantification of 20E, TU, and PA.

Concentration (μg/mL)	Mean (μg/mL ± SD)	Recovery%	CV%
**Turkesterone**			
40	40.06 ± 0.09	100.16	0.53
25	25.22 ± 0.08	100.86	0.75
10	9.94 ± 0.01	99.77	0.24
**20-Hydroxyecdysone**			
40	40.22 ± 0.11	100.54	0.60
25	25.04 ± 0.04	100.18	0.36
10	9.97 ± 0.02	99.77	0.18
**Ponasterone A**			
40	40.31 ± 0.07	100.77	0.39
25	25.10 ± 0.04	100.40	0.37
10	9.96 ± 0.01	99.55	0.30

**Table 3:** Evaluation of the precision of the developed HPLC-PDA method for the detection and quantification of 20E, TU, and PA.

Concentration (μg/mL)	Intra-Day Precision			Inter-Day Precision		
	Mean (μg/mL ± SD)	SEM	CV%	Mean (μg/mL ± SD)	SEM	CV%
**Turkesterone**						
40	40.22 ± 0.51	0.01	1.17	40.30 ± 0.13	0.01	0.32
25	25.08 ± 0.30	0.01	1.18	25.08 ± 0.17	0.01	0.66
10	9.94 ± 0.02	0.01	0.25	10.01 ± 0.13	0.01	1.34
**20-Hydroxyecdysone**						
40	40.22 ± 0.09	0.04	0.23	40.06 ± 0.11	0.04	0.27
25	25.06 ± 0.15	0.07	0.62	25.17 ± 0.19	0.08	0.77
10	9.98 ± 0.05	0.02	0.54	10.00 ± 0.07	0.03	0.72
**Ponasterone A**						
40	40.53 ± 0.29	0.12	0.73	40.61 ± 0.43	0.17	1.06
25	24.99 ± 0.09	0.04	0.37	25.08 ± 0.17	0.08	0.66
10	9.96 ± 0.04	0.01	0.39	9.94 ± 0.04	0.01	0.36

## Data Availability

All relevant data are within the manuscript.

## Supplementary Materials

The following supporting information can be downloaded at: https://www.mdpi.com/article/10.3390/ijms26083739/s1.

## Abbreviations

The following abbreviations are used in this manuscript:

20E	20-Hydroxyecdysone
HPLC	High-performance liquid chromatography
HPLC-PDA	High-performance liquid chromatography with photodiode array detection
LD	Limit of detection
LQ	Limit of quantification
NMG	Nematode growth medium
PA	Ponasterone A
RCE	Rhaponticum carthamoides extract
TU	Turkesterone
