# Supplementary material for: Maral Root Extract and Its Main Constituent 20-Hydroxyecdysone Enhance Stress Resilience in Caenorhabditis elegans
Source: Int J Mol Sci. 2025 Apr 15;26(8):3739. doi: 10.3390/ijms26083739 (PMC12027862; doi:10.3390/ijms26083739)
Supplement: Supplementary file 1 [file ijms-26-03739-s001.zip › ijms-3566861-supplementary.pdf]

SUPPORTING INFORMATION

Figure S1. Resulting calibration curves of target analytes – 20E, TU and PA.

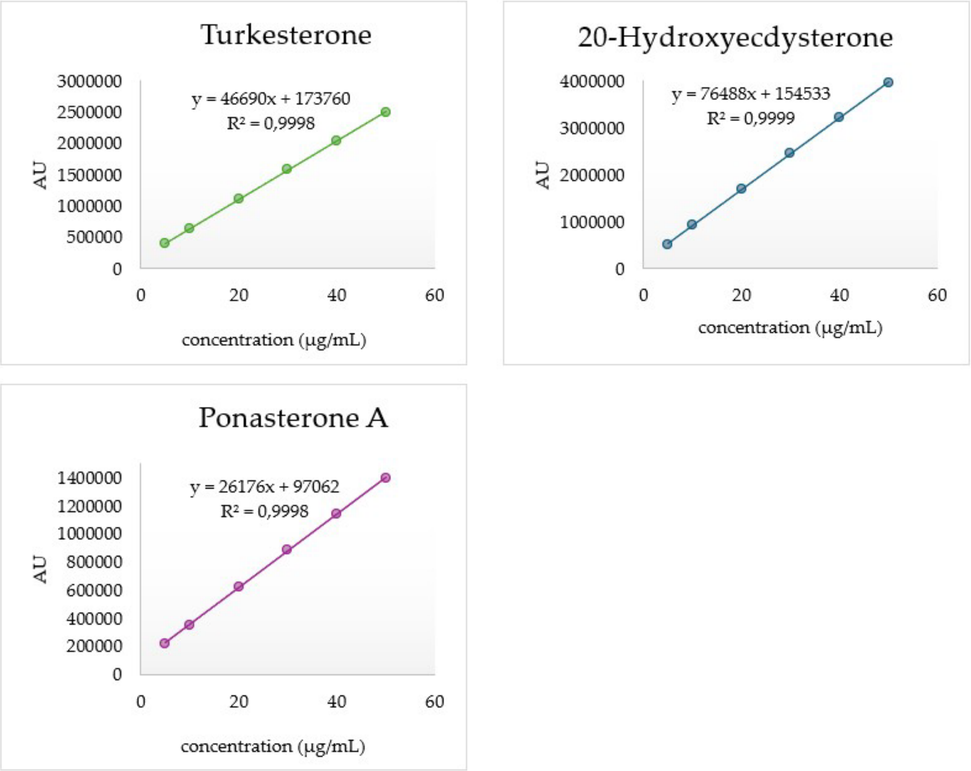

Table S1. Robustness data of the developed HPLC-PDA method for quantification of TU, 20E and PA.

Temperature (°C)	Retention time (min)	Resolution
	**Turkesterone**	
42	5.35	–
45	5.38	–
48	5.41	–
	**20-Hydroxyecdysone**	
42	6.35	4.72
45	6.38	4.69
48	6.37	4.49
	**Ponasterone A**	
42	8.59	9.54
45	8.63	9.58
48	8.56	9.23

Table S2. Stability evaluation of individual compounds stored at 2-8°C for one week, determined at 25 μg/mL (n=3).

Mean concentration (μg/mL±SD)	Recovery%	CV%
**Turkesterone**		
24.82±0.50	98.40	1.99
**20-Hydroxyecdysone**		
25.08±0.09	100.17	0.36
**Ponasterone A**		
24.82±0.20	98.89	0.81

Table S3. Concentrations of 20E, TU, and PA in RCE treatments applied to *C. elegans*.

RCE treatments (μg/mL)	20-Hydroxyecdysone (ng/mL)	20-Hydroxyecdysone (μM)	Turkesterone (ng/mL)	Turkesterone (μM)	Ponasterone A (ng/mL)	Ponasterone A (μM)
10	29	0.060	17	0.034	13	0.027
25	72.5	0.151	42.5	0.086	32.5	0.069
50	145	0.302	85	0.171	65	0.139
100	290	0.603	170	0.342	130	0.279
200	580	1.207	340	0.685	260	0.559
